# Perinatal outcomes during wartime: a multicenter retrospective cohort study in Israel, 2022–2024

**DOI:** 10.1186/s12884-025-08330-4

**Published:** 2025-10-29

**Authors:** Roy Bitan, Racheli Magnezi, Inbal Reuveni, Assaf Tripto, Orly Weinstein, Uri Amikam

**Affiliations:** 1 Department of Management, Health Systems Management Program, Bar Ilan University, Ramat Gan, Israel; 2 Gray Faculty of Medicine, Tel Aviv University, Tel Aviv, Israel; 3 Lis Hospital for Women’s Health, Tel Aviv Sourasky Medical Center, Tel Aviv, Israel; 4 Psychiatric Division, Tel Aviv Sourasky Medical Center, Tel Aviv, Israel; 5 Be’eri Clinic, Clalit Health Services, Ramat-Gan, Israel; 6 Clalit Health Services, Tel-Aviv, Israel

**Keywords:** Wartime obstetrical outcomes, Adverse perinatal outcomes, Premature rupture of membranes, Gestational diabetes, Post-partum hemorrhage

## Abstract

**Background:**

Armed conflicts disrupt healthcare services and expose pregnant women to significant psychological stress, potentially increasing adverse pregnancy outcomes. This study aimed to assess the impact of wartime on pregnancy and birth outcomes amongst Israeli parturients.

**Methods:**

A retrospective cohort study analyzing deliveries at seven university-affiliated hospitals between October 7, 2022, and April 7, 2024. The cohort was divided into two groups: the conflict-exposed group (study group, consisting of women who delivered during the approximately six months following October 7, 2023) and the control group (women who delivered during the same period the previous year). Data extracted from electronic medical records included maternal demographics, comorbidities, and obstetric and neonatal outcomes. Statistical analyses included effect size calculations and multivariable logistic regression, adjusting for relevant maternal confounders.

**Results:**

A total of 30,868 births were included, with 15,384 in the study group and 15,484 in the control group. No significant differences were observed in maternal demographics or comorbidities. The conflict-exposed group had higher rates of premature rupture of membranes (PROM) (15.4% vs. 11.5%, *P* < 0.001, respectively), preterm premature rupture of membranes (PPROM) (3.4% vs. 3%, *P* = 0.022, respectively), gestational diabetes mellitus (GDM) (10.8% vs. 10.0%, *P* = 0.018, respectively), and postpartum hemorrhage (PPH) (4.4% vs. 3.6%, *P* = 0.001, respectively), compared to the control group. The analysis revealed no significant differences in the rates of preterm births and neonatal birth weight below 2,500 g. Effect size analyses and multivariable regression confirmed these associations, demonstrating that conflict exposure was independently linked to increased risks of PROM, PPROM, GDM, and PPH.

**Conclusions:**

Exposure to wartime conditions is associated with an increased risk of various pregnancy and delivery complications, including GDM, PPROM, and PPH. These findings highlight the need for further research into the impacts of war-related stress on pregnancy outcomes and underscore the importance of providing psychological and medical support during wartime.

## Introduction

War and armed conflict have significant implications for public health, resulting in various consequences [1–4]. Previous studies have described a higher incidence of mental disorders among civilians, including anxiety and mood disorders [5, 6]. Moreover, interrupted treatments and delayed diagnoses in conflict zones worsen conditions such as cancer, diabetes, and other chronic illnesses [7]. Among the vulnerable populations affected by conflict, pregnant women and their newborns encounter unique challenges that can significantly influence perinatal outcomes [8]. The disruption of healthcare services, along with increased stress, can negatively impact maternal and neonatal health [8–10]. Evidence from the COVID-19 pandemic in Israel has also demonstrated how large-scale health crises may challenge obstetric care [11].

Pregnancy and delivery outcomes during war and times of conflict have previously been studied [12–15]. Previous studies have shown that maternal exposure to military-related stress correlates with adverse pregnancy outcomes, such as increased risks of preterm birth (PTB), defined as delivery before 37 weeks of gestation, and low birth weight (LBW), which is defined as a birthweight below 2,500 g [13, 16]. When experienced in early pregnancy, military-related acute stress is associated with PTB, while chronic stress in the third trimester correlates with large for gestational age, defined as birthweight above the 90th percentile, and gestational diabetes mellitus (GDM) [12].

When investigating the impact of military-related stress on pregnancy outcomes, previous studies have focused primarily on PTB and low neonatal birth weight and have reported inconsistent findings [13, 17, 18]. One such mixed finding pertains to the risk of PTB in women exposed to life-threatening stressful situations. While some studies have described an increase in the incidence of PTB during wartime [13, 19], others have not found such evidence [20, 21]. Furthermore, previous studies demonstrated conflicting results regarding the incidence of LBW during wartime. While one study did not demonstrate an increase in the incidence of LBW during war [17], others did find such a finding [13, 18]. Additionally, other perinatal outcomes besides the incidence of LBW and PTB have been studied to a lesser extent [13, 16]. Some important obstetric outcomes, such as preterm premature rupture of membranes (PPROM), placental abruption, and mode of delivery, were studied in only a few studies [17, 19, 20].

To address these limitations and highlight the urgent need for further obstetric research, we sought to assess various obstetrical outcomes within the Israeli population during the armed conflict that commenced in October 2023.

## Methods

### Study design

This retrospective cohort study aimed to assess the impact of war on obstetric and neonatal outcomes. The study utilized electronic medical records (EMRs) from seven university-affiliated medical centers that provide healthcare across Israel, including Rabin Medical Center, Meir Medical Center, HaEmek Medical Center, Carmel Medical Center, Soroka Medical Center, Kaplan Medical Center, and Yoseftal Medical Center. The EMRs data were collected from October 7, 2022, to April 7, 2024. The study cohort was divided into two groups: the conflict-exposed group (study group), comprising women who gave birth during the approximately six months after the war in Israel began on October 7, 2023 (October 7, 2023 – April 7, 2024); and the control group, consisting of women who delivered during the corresponding period of the previous year (October 7, 2022 – April 7, 2023). Excluded from the analysis were women giving birth before 24 weeks (below the viability threshold as defined in the position paper of the Israeli Neonatal and Obstetrics and Gynecology Societies [22, 23]); women giving birth after 43 weeks (as Israeli guidelines recommend induction before 42 weeks of gestation [24], making such cases rare and probably an error in the electronic charts); cases involving perinatal mortality; and women who delivered during both of the study periods, such that each woman was included only once in the analysis.

Data extracted from the EMRs included: maternal demographics, comorbidities, and obstetrical and neonatal outcomes. Maternal demographic variables included: age (categorized as under 25 years, 25 to 40 years, and over 40 years); socioeconomic status (classified into five levels ranging from very low to very high, based on the patient’s residential address); and ethnicity (categorized as Jewish or Arab). Pre-existing maternal comorbidities were classified according to ICD-9 codes and included hypothyroidism, pregestational diabetes, malignancy diagnosis before pregnancy, dyslipidemia, and chronic hypertension. Obstetric and neonatal data included PTB (defined as delivery before 37 + 0 weeks of gestation) and gestational age at delivery (categorized as 24 to 31 + 6 weeks, 32 to 33 + 6 weeks, 34 to 36 + 6 weeks, and 37 weeks or more). PTB was defined as the primary outcome of the study. Secondary outcomes included mode of delivery (including cesarean delivery, instrumental delivery, or spontaneous vaginal delivery), the occurrence of premature rupture of membranes (PROM) (defined as rupture of membranes occurring before the start of contractions after 37 weeks of pregnancy), and PPROM (defined as rupture of membranes before 37 + 0 weeks of pregnancy), GDM (defined as screening with a 50 g glucose challenge test at 24–28 weeks, followed by a 100 g oral glucose tolerance test when indicated, with diagnosis made if at least two values met or exceeded the Carpenter–Coustan thresholds) [25], hypertensive disorders of pregnancy (HDP), placental abruption, postpartum hemorrhage (PPH) (defined as either excessive postpartum bleeding with clinical symptoms/signs of hypovolemia, or an estimated blood loss ≥ 1000 mL within the first 24 h after delivery) [26], and obstetric anal sphincter injuries (OASI). Neonatal data included birthweight, LBW (defined as birthweight below 2,500 g), and neonatal Apgar scores at one and five minutes of life.

### Statistical analysis

Statistical analyses were conducted using descriptive and inferential methods. Continuous variables were summarized as means with standard deviations for normally distributed data. Categorical variables were presented as frequencies and percentages. Statistical comparisons were performed using T-tests for normally distributed numerical variables, Mann-Whitney U tests for non-normally distributed numerical variables, and Chi-square tests for categorical variables. Effect sizes were calculated for all maternal and neonatal outcomes, including absolute risk differences (ARD), risk ratios (RR) with 95% confidence intervals (CI), and corresponding p-values. Additionally, multivariable logistic regression analyses were performed to adjust for potential confounders (maternal age, parity, fetal sex, socioeconomic status, ethnicity, and chronic hypertension or pregestational diabetes). Adjusted odds ratio (aOR) with 95% CI and p-values were reported for the main outcomes: PTB, PROM, PPROM, GDM, PPH, and LBW.

All statistical analyses were performed using SAS Enterprise Guide version 8.3 (SAS Institute Inc., Cary, NC, USA). The study was approved by the local institutional review board (IRB number 0117-23-COM2). Informed consent was waived due to the retrospective nature of the study and the use of anonymized medical records with no identifiers.

## Results

**Fig. 1:**
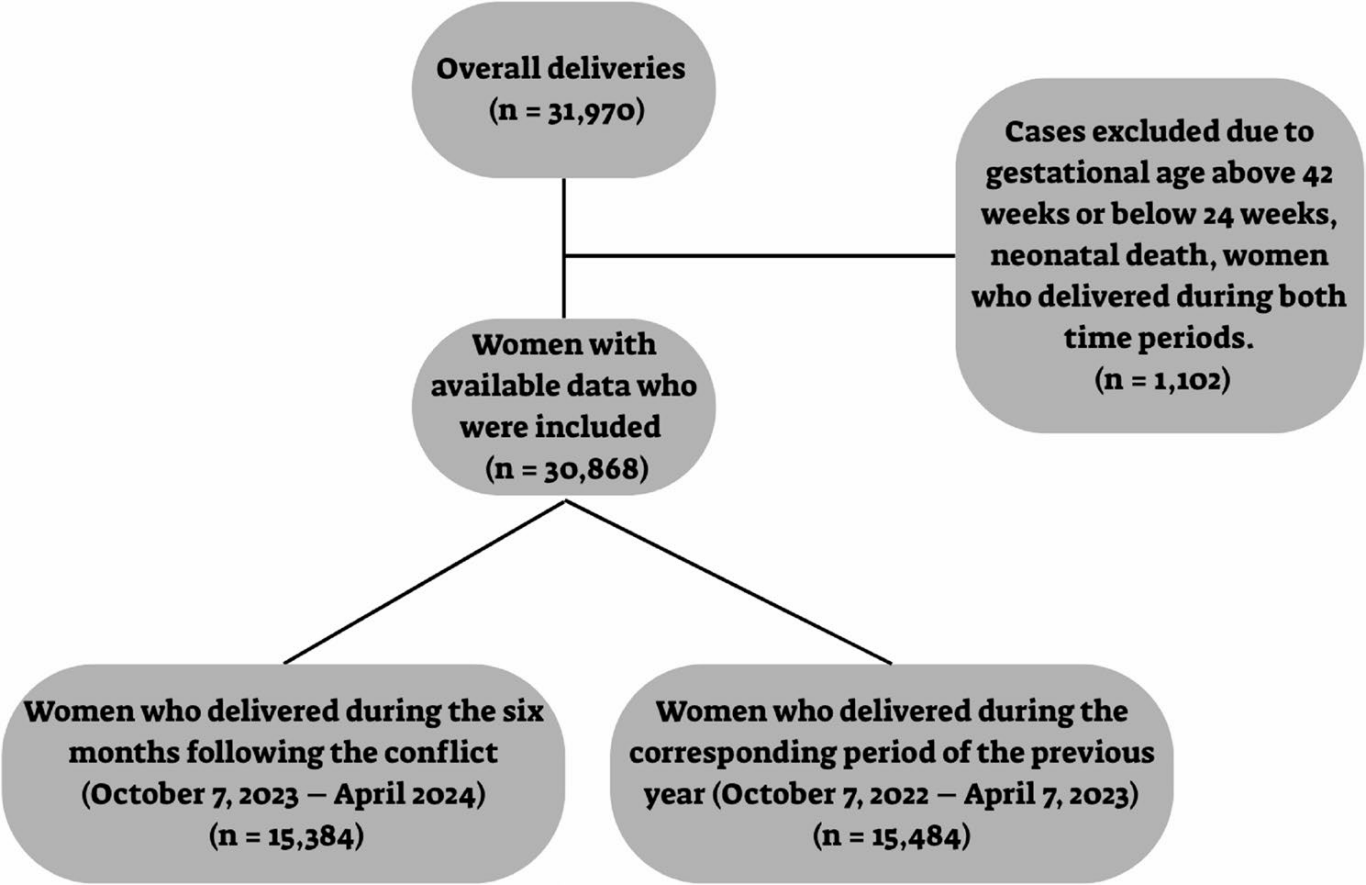
Flowchart of the study participants

The initial dataset included 31,970 deliveries, with 1,102 excluded according to predefined exclusion criteria. Consequently, the total sample consisted of 30,868 deliveries, divided into 15,384 constituting the conflict-exposed group and 15,484 constituting the control group (Fig. 1). Maternal demographic characteristics were similar between groups, with no statistically significant differences in age distribution, socioeconomic status, or ethnicity (*p* > 0.05 for all comparisons), except for parity, which was significantly lower in the conflict-exposed group than in the control group (median parity: 1 [Interquartile range (IQR) 0–2] versus 1 [IQR 1–3], *P* < 0.001, respectively). There were no differences between groups in maternal comorbidities, including hypothyroidism, malignancy, lipid disorders, chronic hypertension, and pre-gestational diabetes mellitus (Table 1).

**Table 1:** Maternal demographics & comorbidities

			Conflict-exposed group (*n* = 15,384)	Control group (*n* = 15,484)		*P*-value
Age, mean (SD), years			30.2 *±* 5.6	30.3 *±* 5.6		0.321
Age, n (%)	< 25 years		3296 (21.4)	3303 (21.3)		0.720
	25 to 40 years		11,574 (75.2)	11,638 (75.2)		
	> 40 years		514 (3.3)	543 (3.5)		
Parity, median (IQR)			1 (0–2)	1 (1–3)		< 0.001
Socioeconomic level, n (%)		Very low	2332 (17.5)	2287 (17.0)		0.272
		Low	3285 (24.7)	3332 (24.7)		
		Medium	3688 (27.7)	3672 (27.3)		
		High	3111 (23.4)	3298 (24.5)		
		Very high	884 (6.6)	884 (6.6)		
Fetal sex, n (%)		Female	7467 (48.5)	7503 (48.5)		0.918
		Male	7920 (51.5)	7980 (51.5)		
Ethnicity, n (%)		Arab	4948 (52.6)	5016 (52.9)		0.612
		Jewish	4463 (47.4)	4458 (47.1)		
Maternal comorbidities, n (%)		Hypothyroidism	1224 (8.0)		1216 (7.9)	0.737
		Malignancy	375 (2.4)		336 (2.2)	0.117
		Lipid disorders	539 (3.5)		559 (3.6)	0.613
		Chronic hypertension	205 (1.3)		204 (1.3)	0.907
		Pregestational diabetes	244 (1.6)		336 (2.2)	0.117

*Abbreviation* *SD* standard deviation, IQR Interquartile range

Regarding obstetric and delivery outcomes, as presented in Table 2, the primary outcome of PTB did not differ significantly between groups. The study group had higher rates of several secondary outcomes, including PROM (15.4% vs. 11.5%, *P* < 0.001, respectively), PPROM (3.4% vs. 3%, *P* = 0.022, respectively), GDM (10.8% vs. 10%, *P* = 0.018, respectively), and PPH (4.4% vs. 3.6%, *P* = 0.001, respectively). No statistically significant differences were observed in other obstetric or neonatal outcomes, such as placental abruption, gestational age at delivery, mode of delivery, OASI, HDP, neonatal birth weight, LBW, and Apgar score. Effect size analyses are shown in Table 3.

**Table 2:** Obstetric and neonatal outcomes

			Conflict-exposed group (*n* = 15,384)	Control group (*n* = 15,484)	*P*-value
Gestational complications	GDM, n (%)		1661 (10.8)	1542 (10.0)	0.018
	HDP, n (%)		637 (4.1)	635 (4.1)	0.860
	PROM, n (%)		2368 (15.4)	1783 (11.5)	< 0.001
	PPROM, n (%)		524 (3.4)	457 (3.0)	0.022
	Placental abruption, n (%)		127 (0.8)	107 (0.7)	0.173
Intrapartum outcomes	Gestational age at delivery, mean (SD), weeks + days		38 + 6(*±* 1 + 3)	38 + 6(*±* 1 + 3)	0.244
	PTB, n (%)		758 (4.9)	747 (4.8)	0.672
	PTB groups, n (%)	24 to 31 + 6 weeks	55 (0.3)	56 (0.3)	0.698
		32 to 33 + 6 weeks	162 (1.1)	139 (0.9)	
		34 to 36 + 6 weeks	541 (3.5)	552 (3.6)	
	Birth type, n (%)	Vaginal delivery	10,243 (78.5)	10,395 (78.6)	0.066
		Assisted vaginal delivery (vacuum or forceps)	593 (4.5)	531 (4.0)	
		CD	2205 (16.9)	2306 (17.4)	
	OASI, n (%)		10 (0.1)	9 (0.1)	0.988
Peripartum/postpartum outcomes	PPH, n (%)		672 (4.4)	564 (3.6)	0.001
	Birthweight, mean (SD), gram		3246.7 *±* 460.3	3245.9 *±* 457.7	0.876
	LBW, n (%)		778 (5.1)	767 (5.0)	0.676
	Apgar 1 min, mean (SD)		8.9 *±* 0.7	8.9 *±* 0.8	0.820
	Apgar 5 min, mean (SD)		9.9 *±* 0.4	9.9 *±* 0.4	0.339
	Apgar 5 min > 7, n (%)		14,955 (99.8)	15,104 (99.8)	0.594

*Abbreviations GDM* gestational diabetes mellitusk,* HDP* hypertensive disorders of pregnancy,* PROM* premature rupture of membranes, *PPROM* preterm premature rupture of membranes, *OSAI* obstetric anal sphincter injuries, *SD* standard deviation, *PTB* preterm birth *CD* cesarean delivery, *PPH* post-partum hemorrhage, *LBW* low birthweight

**Table 3:** Effect sizes for primary obstetric and neonatal outcomes

		Conflict-exposed group (*n* = 15,384)	Control group (*n* = 15,484)	ARD	RR (95% CI)	*P*-value
GDM, n (%)		1555 (10.65)	1450 (9.83)	+ 0.82	1.09 (1.02–1.17)	0.015
HDP, n (%)		594 (4.07)	596 (4.04)	+ 0.03	1.01 (0.90–1.13)	0.920
PROM, n (%)		2246 (15.38)	1713 (11.61)	+ 3.77	1.33 (1.25–1.42)	< 0.001
PPROM, n (%)		490 (3.36)	426 (2.89)	+ 0.47	1.16 (1.01–1.33)	0.034
Placental abruption, n (%)		121 (0.84)	101 (0.68)	+ 0.14	1.21 (0.93–1.58)	0.154
PTB, n (%)		715 (4.90)	707 (4.80)	+ 0.1	1.01 (0.90–1.12)	0.750
PTB groups, n (%)	24 to 31 + 6 weeks	51 (0.35)	52 (0.35)	0	0.99 (0.67–1.46)	1
	32 to 33 + 6 weeks	154 (1.06)	134 (0.91)	+ 0.15	1.16 (0.92–1.46)	0.224
	34 to 36 + 6 weeks	510 (3.49)	521 (3.53)	−0.04	0.99 (0.88–1.12)	0.887
Birth type, n (%)	Vaginal delivery	9708 (78.48)	9926 (78.68)	−0.2	1.00 (0.98–1.01)	0.706
	Assisted vaginal delivery (vacuum or forceps)	572 (4.62)	512 (4.06)	+ 0.57	1.14 (1.01–1.28)	0.031
	CD	2090 (16.9)	2174 (17.23)	−0.34	0.98 (0.93–1.04)	0.489
OASI, n (%)		10 (0.07)	9 (0.06)	+ 0.01	1.12 (0.46–2.76)	0.801
PPH, n (%)		635 (4.35)	542 (3.67)	+ 0.68	1.18 (1.06–1.32)	0.003
LBW, n (%)		740 (5.07)	721 (4.89)	+ 0.18	1.04 (0.94–1.15)	0.473
Apgar 5 min < 7, n (%)		34 (0.24)	30 (0.21)	+ 0.03	1.15 (0.70–1.88)	0.620

*Abbreviations ARD* absolute risk difference,* RR* risk ratio, *CI* confidence interval *GDM* gestational diabetes mellitus, *HDP* hypertensive disorders of pregnancy, *PROM* premature rupture of membranes *PPROM* preterm premature rupture of membranes, *PTB* preterm birth, *CD* cesarean delivery, *OSAI* obstetric anal sphincter injuries, *PPH* post-partum hemorrhage *LBW* low birthweight

All the univariate associations found to be significant between war exposure and obstetrical complications were also significant in the multivariable logistic regression analyses (Table 4), including PROM (aOR 1.34, 95% CI 1.26–1.43), PPROM (aOR 1.15, 95% CI 1.02–1.30), GDM (aOR 1.11, 95% CI 1.02–1.20), and PPH (aOR 1.15, 95% CI 1.02–1.30), with no significant differences observed for PTB and LBW.

**Table 4:** Multivariable regression analysis for main outcomes

	aOR	CI 95%	*P*-value
GDM	1.111	1.024–1.204	0.011
PROM	1.341	1.260–1.427	< 0.001
PPROM	1.152	1.024–1.295	0.018
PTB	1.027	0.923–1.143	0.6213
PPH	1.151	1.023–1.295	0.019
LBW	1.015	0.913–1.128	0.783

*Abbreviations aOR* adjusted odds ratio, *CI* confidence interval, *GDM* gestational diabetes mellitus *PROM* premature rupture of membranes, *PPROM* preterm premature rupture of membranes, *PTB* preterm birth, *PPH* post-partum hemorrhage, *LBW* low birthweight

## Discussion

Our study aimed to evaluate perinatal outcomes during wartime. Our key results were that maternal exposure to war conditions during pregnancy was associated with a higher incidence of PPROM, GDM, and PPH.

Several hypotheses exist for the adverse outcomes observed in the wartime group. The primary hypothesis is that women experience heightened stress levels during times of conflict. Numerous mechanisms have been suggested to elucidate this association, with the most widely accepted theory grounded in the hypothalamic–pituitary–adrenal (HPA) axis response to stress. The activation of the HPA axis, a component of the stress response, culminates in the elevated secretion of cortisol and catecholamines, which subsequently increases blood pressure, elevated blood glucose levels, and persistent oxidative stress [27–30]. Secondly, health services are severely disrupted during times of war, both in terms of the services provided and due to the shortage of healthcare resource providers [31, 32]. Lastly, during wartime, women may have fewer medical encounters, which can hinder pregnancy outcomes [8, 15].

In our cohort, there were higher rates of rupture of membranes (ROM), both PPROM and PROM, in the study group, compared to the control group. This increase was statistically significant among women delivering at term and during the preterm period. These may hypothetically relate to higher levels of stress experienced by women who were pregnant and gave birth during wartime. As suggested by numerous previous studies [33–36], cortisol hormone levels and the HPA axis can potentially mediate the link between stress and both ROM and PTB. Cortisol can affect the immune response, inflammation, and uterine contractions, which are associated with the onset of labor [28, 37–40]. Notably, the rate of PPROM in our study was 3% before the war, consistent with previously published data [41], and increased to 3.4% during wartime, which was found to be significantly higher.

We found an increased incidence of GDM among women in the conflict-exposed group. This finding contradicts previous studies’ findings [13, 17]. Several potential explanations could explain this. First, the association between high-stress levels and increased blood sugar levels is well-documented in the literature, with high-stress levels linked to an elevated risk of GDM [42, 43]. Additionally, during stressful times, people can experience emotional eating, consuming more high-calorie, energy-dense foods when under stress, while simultaneously reducing their physical activity levels due to fatigue, anxiety, or lack of motivation. Both factors have been found to be linked to obesity and GDM [44–46]. It should be noted that GDM usually develops during earlier stages of pregnancy [47–49]. Therefore, its incidence is likely influenced more by exposures and conditions in the first and second trimesters, which may not have been fully captured in the current study design.

The present study also revealed increased PPH rates in the conflict-exposed group. This can be attributed to several factors. First, the delivery room and maternity ward were probably understaffed due to drafting to the military reserves. Additionally, it is plausible that baseline hemoglobin levels were lower among women in the conflict-exposed group, as they may have had reduced access to antenatal care during pregnancy and in the immediate period prior to delivery. This could have led to a higher prevalence of undiagnosed or untreated antepartum anemia, thereby increasing susceptibility to clinically significant blood loss during delivery. Furthermore, the higher rates of GDM, PPROM, and PROM in the study group could have also contributed to the increased incidence of PPH, all of which are generally recognized as risk factors for PPH [41, 50]. Further research is needed to determine whether the increased rate of PPH in the study group is related to a shortage and changes in medical staffing, linked to the increased rates of ROM, or caused by other factors not clarified in the current study.

Interestingly, the incidence of PTB in our study was similar between the study and control groups. A systematic review by Keasley et al. [10] reported mixed findings, with some studies—such as those conducted in Iraq and Libya—supporting an association between conflict exposure and increased rates of PTB [51, 52], while others, particularly those from Bosnia, did not demonstrate the same effect [20, 21]. There could be several explanations for this finding. Firstly, the overall incidence of PTB in both groups during our study was less than 5%, which is lower than the PTB rate reported in Israel, which was 6.5% in 2022 [53]. Additionally, this finding may be explained by the fact that women in our study come from various areas in Israel. Hypothetically, women living closer to the border, who are more affected by the war and express higher levels of stress, may exhibit higher rates of PTB than those living farther away. Furthermore, even though our cohort consisted of more than 30,000 women, it may be underpowered to detect a difference in the incidence of PTB between the two groups.

The rate of LBW infants was comparable between the two groups in our study. Previous studies did find a higher incidence of LBW infants in the war-exposed group [10, 13, 18, 54], except for one study from Israel [17]. A systematic review which included data from over one million pregnancies across various global conflicts, found consistent evidence linking maternal exposure to armed conflict with an increased risk of LBW, primarily due to prenatal stress and impaired access to healthcare [10]. The lack of such an association in our study could be explained by the relatively short observation period of approximately six months for each group. It is possible that stress-related fetal growth restriction requires exposure during the early stages of pregnancy, particularly the first and second trimesters, and our study period may not have captured such timing. Furthermore, this phenomenon may be ascribed to an inadequately low level of mental stress arising from this military conflict.

The biological mechanisms underlying the association between stress and adverse pregnancy outcomes, such as intrauterine growth restriction, early gestational age at birth, PTB, small-for-gestational-age, and LBW, involve a complex interplay of various factors. Among these factors are hormonal changes, inflammatory responses, altered immune function, and neuroendocrine signaling, including the release of stress hormones (primarily cortisol) [28, 37, 38, 55–58]. Additionally, the impact of stress may extend beyond direct physiological effects, affecting healthcare access, prenatal care utilization, and maternal behaviors during pregnancy. The disruption of routine medical services and heightened emotional distress during wartime could further exacerbate stress-related risks. Understanding these pathways is crucial for developing targeted interventions to mitigate the adverse effects of stress on pregnancy and improve maternal and neonatal health outcomes in conflict-affected regions.

The present study examined a variety of adverse pregnancy outcomes in the war-affected population, thus contributing to a broader understanding of pregnancy outcomes during times of war. To assess a broad range of obstetric outcomes, this research examined a large cohort of women exposed to wartime stress. Selecting the cohort of women from seven hospitals throughout Israel enhanced the generalizability of our results to the entire Israeli population. Additionally, the control group consisted of parturients who delivered during the equivalent period in the previous year, thus minimizing seasonal and protocol-related confounders. By comparing perinatal outcomes in the conflict-exposed group to those in the control group, we gained valuable insights into the direct impact of wartime on pregnancy and delivery outcomes.

Though providing important insights into the adverse effects of war-related stress on obstetric outcomes, our study is not devoid of limitations. Firstly, it is limited by its retrospective design and reliance on electronic medical records, which may contain incomplete data. Another limitation is the potential variation in exposure levels to the war’s direct and indirect effects, which were not quantified in our study. Pregnant individuals living in high-conflict areas may have experienced more pronounced stress responses compared to those in safer regions. Further, reliance on hospital records without thorough maternal psychological evaluations may have restricted our capacity to establish causality and quantify the degree of stress and adverse perinatal outcomes. The overall incidence of OASI appeared lower than expected, likely reflecting underdiagnosis and underreporting in the records [59]. In addition, cases of perinatal mortality, including intrauterine fetal demise, were excluded from the analysis due to substantial variability in definitions across centers and the inability to validate cases individually because of patient anonymity.

Future research into the impact of war-related stress on perinatal outcomes may be necessary to address these limitations.

## Conclusion

Our findings indicate that pregnancy and delivery during wartime were associated with increased risks of PPROM, GDM, and PPH, while demonstrating no statistically significant association with PTB or LBW. These results highlight the complexity of potential war-related stress effects on pregnancy, emphasizing the need for further investigation into protective factors and intervention strategies. Ensuring comprehensive prenatal care and psychological support during wartime remains critical for mitigating potential adverse effects on maternal and neonatal health.

## Data Availability

The datasets used and/or analyzed during the current study are available from the corresponding author upon reasonable request.

## Abbreviations

PTB: Preterm birth
LBW: Low birth weight
GDM: Gestational diabetes mellitus
PPROM: Preterm premature rupture of membranes
EMR: Electronic medical record
ICD-9: International classification of diseases, ninth revision
PROM: Premature rupture of membranes
HDP: Hypertensive disorders of pregnancy
PPH: Postpartum hemorrhage
OASI: Obstetric anal sphincter injuries
ARD: Absolute risk difference
RR: Risk ratio
CI: Confidence interval
aOR: Adjusted odds ratio
IRB: Institutional review board
IQR: Interquartile range
HPA: Hypothalamic–pituitary–adrenal
ROM: Rupture of membranes
